# Le syndrome des jambes sans repos: fréquence et facteurs de risque chez l'hémodialysé

**DOI:** 10.11604/pamj.2015.20.29.5723

**Published:** 2015-01-13

**Authors:** Illiassou Soumeila, Salia Keita, Anis Elhassani, Mohamed Sidibé, Khadija Alaoui, Nadia Kabbali, Mohamed Arrayhani, Tarik Sqalli

**Affiliations:** 1 Service de Néphrologie, CHU Hassan II, Fès, Maroc; 2 Service d'Epidémiologie et de Recherche Clinique, Faculté de Médecine et de Pharmacie, Fès, Maroc; 3 Equipe de Recherche REIN, Faculté de Médecine et de Pharmacie, Fès, Maroc

**Keywords:** Hémodialysé, SJSR, facteurs de risque, Hemodialysis, restless legs syndrome, risk factor

## Abstract

Le syndrome des jambes sans repos (SJSR) ou syndrome d'impatience musculaire est un trouble moteur caractérisé par des sensations désagréables dans les jambes. Les causes sont mal connues et sa fréquence est estimée entre 25% et 75% chez les hémodialysés. Il s'agit d'une étude transversale monocentrique menée au centre d'hémodialyse du CHU Hassan II de Fès (hôpital Al Ghassani) entre décembre 2012 et janvier 2013. Nous avons défini le syndrome de jambes sans repos selon la définition de l'international restless legs study group de 2003 reposant sur 4 critères essentiels au diagnostic. L'international restless legs syndrome scale (IRLES) a été coté par un même néphrologue pour mesurer la sévérité du syndrome des jambes sans repos. 84 hémodialysés ont répondu au questionnaire avec 41,7% de cas de SJSR dont 6,6% de formes graves. Nous avons retrouvé une association entre le SJSR et la carence martiale p(0,018), la néphropathie initiale p(0,041), l'HTA p(0,026) et le sexe féminin p(0,024). Dans notre série, il ressort que la carence martiale et l'HTA sont les principaux facteurs de risque modifiables de ce syndrome chez nos patients. Les facteurs traditionnels comme le tabagisme, l’âge supérieur à 50 ans et la dialyse inadéquate ne sont pas associés à ce trouble dans notre série.

## Introduction

Le syndrome des jambes sans repos (SJSR) est un trouble sensitivo-moteur. Le patient rapporte une sensation pénible (impatiences, paresthésies, parfois douleurs ou brûlures), le plus souvent localisée aux membres inférieurs et bilatérale, associée à un besoin impérieux de bouger les jambes, survenant particulièrement au repos et le soir [1, 2] Le véritable père du syndrome éponyme est le neurologue suédois Ekbom, en 1944, qui en fait une description précise, le baptise «restless legs syndrome», collige 34 cas et estime même sa prévalence [3]. La physiopathologie du syndrome est incomplètement élucidée. Il est surtout primaire, fait intervenir une composante génétique très probable (plus des 2/3 des cas sont familiaux) et ou un dysfonctionnement dopaminergique central, possiblement sur la voie hypothalamo-spinale [4]. Il peut être secondaire, lié à une dysrégulation du passage transmembranaire du fer, à une insuffisance rénale terminale, une grossesse ou une neuropathie périphérique. La fréquence est variable d'une population à une autre et selon les régions du monde. Elle représente 11% en Europe du nord [5], 8,5% en France [6], entre 5 et 15% à Singapour [7] et en Amérique du nord dans la population générale [8]. La fréquence chez l'hémodialysé varie entre 25% à 57% [9, 10]. Peu d’études ont été réalisées pour déterminer la fréquence du SJSR dans notre population d'hémodialysés et rechercher les facteurs associés au SJSR d'où notre intérêt pour ce sujet.

## Méthodes

Il s'agit d'une étude transversale monocentrique menée chez l'ensemble des patients hémodialysés chroniques adultes du centre d'hémodialyse du CHU Hassan II de Fès (hôpital Al Ghassani) entre décembre 2012 et janvier 2013. Nous avons exclu tous les patients présentant pas une infection récente, un cancer, un alcoolisme abusif, une insuffisance hépatique, une neuropathie sévère. Nous avons défini le syndrome de jambes sans repos selon la définition de l'international restless legs study group de 2003 reposant sur 4 critères essentiels au diagnostic et les critères qui y sont associés (Tableau 1). L'international restless legs syndrome scale (IRLES) a été coté par un même néphrologue pour mesurer la sévérité du syndrome des jambes sans repos. Cet index comprend 10 questions évaluant les symptômes durant les sept derniers jours et d'une manière générale. Chacune des dix questions étant cotée de 0 (inexistant) à 4 (très important). Nous avons défini l'hypertension artérielle (HTA) selon la définition de la société française d'HTA de 2013 qui définit l'HTA comme une PAS ≥ 140 mmHg et/ou PAD ≥90mmHg et dans notre contexte tous les patients sous traitement anti-hypertenseur y ont été inclus [11]. La carence martiale a été définie selon les recommandations des KDIGO 2012 par un taux de ferritine ∠100µg/l [12]. Une analyse univariée a été réalisée pour rechercher les facteurs liés au SDJS. L'analyse par régression logistique multiple a été utilisée pour évaluer les effets de différents facteurs sur la présence du SDJS. Les données ont été saisies sur Microsoft Excel 2007 et l'analyse statistique a été réalisée par le logiciel SPSS 17.0.

**Tableau 1:** Paramètres démographiques et dialytiques.

Paramètres	Résultats (n= 84 HDC)
Sex-ratio (H/F)	0,97
Age (ans)	50 ± 15
Ancienneté en HD (année)	7 ± 4
Kt/V hebdomadaire	1,34 ± 0,22

## Résultats

**Figure 1:**
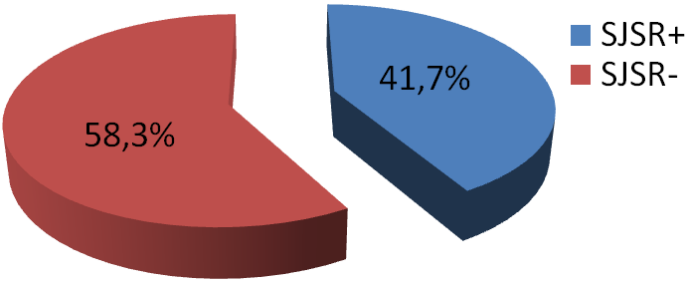
Fréquence du syndrome des jambes sans repos chez les hémodialysés

**Figure 2:**
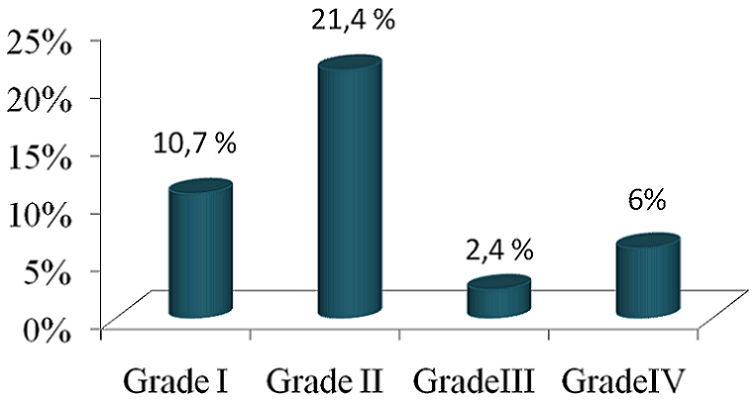
Gradation du SJSR selon la sévérité dans notre série

Notre étude a porté sur 84 Patients hémodialysés avec un âge moyen de 50,55±15,35 ans, un sex-ratio (H/F) de 0,97, une ancienneté d'hémodialyse moyenne de 7,46±4,55 ans et Kt/V moyen à l’équilibre de 1,34±0,22 (Tableau 1). La prévalence du syndrome des jambes sans repos dans notre série est de 41,6% (Figure 1) dont seulement 5 cas de forme très sévère (Figure 2). En analyse univariée, il ressort que le SJRS est lié à la carence martiale p(0,018), le sexe féminin p(0,024) (Tableau 2), la néphropathie vasculaire p(0,041), et l'HTA p(0,026) (Tableau 3). On note par ailleurs une association des troubles du sommeil avec le SJSR p (0.04). En analyse multivariée, seule l'HTA p(0,037) et la carence martiale p(0,025) étaient significativement liées au syndrome des jambes sans repos avec respectivement des odd-ratio de 2,95 et 3,78 (Tableau 4). Les autres paramètres de dialyse, notamment le bilan phospho-calcique n’étaient pas significativement liés au SJSR avec respectivement p(0,64) pour la calcémie et p(0,79) pour la phosphorémie.

**Tableau 2:** Paramètres démographiques et dialytiques liés au SJSR (Résultats de l'analyse univariée)

Paramètres	SJSR-(n = 49)	SJSR+ (n = 35)	p*
Sexe féminin (%)	59,2	65,7	0,024
Age (ans)	50,31±16,39	50,89±50,89	NS
Carence martiale (%)	13	37	0,018
IMC (kg/m2)	21,61± 3,86	21,99± 3,87	NS
Kt/V journalier	1,35 ± 0,23	1,34 ± 0,21	NS
Ancienneté en HD (ans)	7,5 ± 4,7	7,5 ± 4,4	NS
Hémoglobinémie (g/dl)	10,14± 1,99	10,57± 1,58	NS

**Tableau 3:** Facteurs de risque liés au SJSR selon les antécédents du patient (analyse uni variée).

Antécédents	SJSR-(n = 49)	SJSR+ (n = 35)	p*
HTA (%)	25	67	0,026
Diabète (%)	6,2	5,7	NS
Tabagisme (%)	8,2	8,5	NS
Néphropathie initiale: (%)			
Vasculaire	19	37	0.041
Glomérulaire	18	20	NS
NTIC	08	17	NS
Indéterminée	55	26	NS

NTIC: Néphropathie tubulo-interstitielle chronique

**Tableau 4:** Facteurs liés au SJSR (analyse multi variée)

Facteurs de risque	SJSR- n = 49	SJSR+ n = 35	OR (IC = 95%)	p*
HTA (%)	25	67	3,78(1,18-12,08)	0,026
Carence martiale (%)	13	37	2,55 (1,07-8,2)	0,037

## Discussion

Cette étude est la première au Maroc à s'intéresser aux facteurs de risque liés au SJSR dans la population des hémodialysés. La prévalence dans notre série s’élève à 41,7%. La prévalence dans les autres études épidémiologiques réalisées aux Maroc dans la population des hémodialysés est de l'ordre de (46.2%) et opposable à celle représentée dans une série brésilienne (21.5%) [13, 14]. Les formes sévères représentent 6% des cas dans notre série, ce qui est superposable aux 6,6% de la série indienne qui s'est déroulée avant la mise en place des critères de l'IRLES [15]. En général, le syndrome des jambes sans repos prédomine chez la femme (10.5% contre 5.8%) [8]. Dans notre étude, le sexe féminin ressort comme un facteur prépondérant de ce syndrome (65,7% contre 34,3%). Par ailleurs, nous ne retrouvons pas de corrélation entre l’âge, l'ancienneté en hémodialysé, la dose de dialyse et la survenue du SJSR [16, 17].

Les troubles du sommeil p(0,04) sont fréquemment associés au syndrome des jambes sans repos et constituent plus une conséquence qu'une cause de ce dernier. La carence martiale pourrait entrainer le SJSR en diminuant la concentration en fer dans le locus niger et le putamen selon les données de l'IRM [18], diminution corrélée à l′intensité de la symptomatologie. Les autopsies de malades atteints de SJSR mettent en évidence une diminution du fer et de la ferritine des neurones dopaminergiques de la substance noire [19]. Ce déficit en fer serait dû à un défaut d′acquisition du fer par les cellules dopaminergiques de la substance noire; il provoquerait une diminution de l′expression des molécules Thy 1, engendrant ainsi un manque de stabilité des synapses dopaminergiques [19]. Dans notre série, il ressort comme un facteur de risque déterminant de la survenue du SJSR avec un odd-ratio de 2,55 et p(0.03). La présence de l'HTA constitue un des facteurs prédisposant au SJSR ce qui est retrouvé chez la grande majorité des auteurs. Elle serait associée à la sévérité du SJSR et au syndrome d'apnée du sommeil [20]. En analyse univariée, la néphropathie vasculaire semble associée au SJSR du fait de l’étiologie hypertensive de cette dernière.

## Conclusion

Le syndrome des jambes sans repos est un syndrome fréquent et encore sous diagnostiqué chez les hémodialysés. Les facteurs liés à sa survenue sont nombreux et la physiopathologie mal définie. Dans notre série, il ressort que ce syndrome est fréquent dans la population des hémodialysés (41,6%). Les facteurs prédisposants sont la carence martiale et l'HTA. Cependant des traitements prometteurs à base de prégabaline sont l'occasion de vulgariser la connaissance de la pathologie afin d'assurer une prise en charge adéquate.
